# Accuracy of computer-aided image analysis in the diagnosis of odontogenic cysts: A systematic review

**DOI:** 10.4317/medoral.24238

**Published:** 2020-11-28

**Authors:** Marcos Alan Vieira Bittencourt, Pedro Henrique de Sá Mafra, Roxanne Silva Julia, Bruno Augusto Nassif Travençolo, Pedro Urquiza Jayme Silva, Cauane Blumenberg, Virgínia Kelma dos Santos Silva, Luiz Renato Paranhos

**Affiliations:** 1 Department of Pediatric and Community Dentistry, Federal University of Bahia, Salvador, Brazil; 2 School of Denstistry, Federal University of Uberlândia, Minas Gerais, Brazil; 3 School of Computing, Federal University of Uberlândia, Minas Gerais, Brazil; 4 Postgraduate Program in Dentistry, Federal University of Uberlândia, Minas Gerais, Brazil; 5 Postgraduate Program in Epidemiology, Federal University of Pelotas, Rio Grande do Sul, Brazil; 6 Department of Dentistry, Federal University of Sergipe, Lagarto, Brazil; 7 Department of Preventive and Community Dentistry, Federal University of Uberlândia, Minas Gerais, Brazil

## Abstract

**Background:**

This study aimed to search for scientific evidence concerning the accuracy of computer-assisted analysis for diagnosing odontogenic cysts.

**Material and Methods:**

A systematic review was conducted according to the PRISMA statements and considering eleven databases, including the grey literature. Protocol was registered in PROSPERO (CRD 42020189349). The PECO strategy was used to define the eligibility criteria and only studies involving diagnostic accuracy were included. Their risk of bias was investigated using the Joanna Briggs Institute Critical Appraisal tool.

**Results:**

Out of 437 identified citations, five papers, published between 2006 and 2019, fulfilled the criteria and were included in this systematic review. A total of 5,264 images from 508 lesions, classified as radicular cyst, odontogenic keratocyst, lateral periodontal cyst, glandular odontogenic cyst, or dentigerous cyst, were analyzed. All selected articles scored low risk of bias. In three studies, the best performances were achieved when the two subtypes of odontogenic keratocysts (solitary or syndromic) were pooled together, the case-wise analysis showing a success rate of 100% for odontogenic keratocysts and radicular cysts, in one of them. In two studies, the dentigerous cyst was associated with the majority of misclassifications, and its omission from the dataset improved significantly the classification rates.

**Conclusions:**

The overall evaluation showed all studies presented high accuracy rates of computer-aided systems in classifying odontogenic cysts in digital images of histological tissue sections. However, due to the heterogeneity of the studies, a meta-analysis evaluating the outcomes of interest was not performed and a pragmatic recommendation about their use is not possible.

** Key words:**Computer-assisted diagnosis, computer-assisted image analysis, computer-assisted image processing, odontogenic cysts, keratocysts, radicular cysts.

## Introduction

Proper diagnosis of odontogenic cysts is crucial because different biological behaviors of the various types of cysts require different treatment plans, and present significantly different risks for affected patients (1). Cysts in the jaws are comparatively easy to diagnose on the basis of radiographic images, but it is sometimes difficult to differentiate them from odontogenic tumors (2). Evolution of technology has provided some changes in this segment, and the use of digital equipment has already been subject of several studies in multiple countries (3-5). Most research on classification of odontogenic cysts has been based on histopathological features and some clinical considerations (6). Despite the digital revolution on imaging technologies, an accurate diagnosis is still dependent on the experience and judgment of a specialist (7), and this is time consuming and prone to human error (8). Aiming to minimize the subjectivity of personal evaluation and to reduce the workload of oral pathologists (9), many attempts have been made to apply automated machine vision systems using mathematical formulas, image processing, and computational algorithms for the diagnosis of some odontogenic cysts (10).

The computer-aided diagnosis (CAD) was developed to perform an automated or semi-automated microscopy image analysis of a large amount of data, including generalized textural features such as contrast or entropy, and cytological features such as the number, size, and distribution of the cell nuclei or borders (11,12). As CAD is a noninvasive tool that provides instant diagnostic results (13), it has been successfully used in many high-impact clinical areas, supporting medical professionals with a valuable second opinion and being a great complementary tool (14-16). Although common in medicine, the field of dentistry has benefited little from the advancements of machine vision systems. According to Yilmaz *et al*. (17), research conducted on dental images in the field of computer vision is extremely challenging. Since types of odontogenic cysts differ primarily in the structure of the epithelial layer, an automated algorithm for segmentation of the epithelial cells in digital images of hematoxylin and eosin stained samples is crucial to the realization of a fully automated CAD protocol for odontogenic cysts (9).

Landini (18), using a systematic clustering routine, computed various morphometrical parameters extracted from epithelial cells of odontogenic keratocysts and radicular cysts in order to diagnose these lesions. Later, Han *et al*. (19) introduced a machine learning based approach, the cascaded Haar classification, to explore the feasibility of using such algorithms to classify those same lesions. A set of image features and two statistical classifiers, a support vector machine and an ensemble method using logistic regression as the base learner, were proposed by Frydenlund *et al*. (20) for automatically distinguishing between four types of odontogenic cysts. Florindo *et al*. (21) used a machine vision algorithm, the Bouligand-Minkowski descriptors, to classify the epithelial lining of odontogenic keratocysts and radicular cysts. Finally, a deep-learning-based identification using convolutional neural networks was proposed by Sakamoto *et al*. (22) also for diagnosis of odontogenic keratocysts.

All the above-mentioned authors have tried to create computer learning methodologies to automate the task of establishing a histopathological diagnosis for odontogenic cysts. However, as an accurate and fast classification of these lesions is clinically very important, since it would improve the quality of patient care and reduce the workload of professionals, the present systematic review aims to investigate if there is scientific evidence that supports the validity of computer image processing in reliably performing the diagnosis of odontogenic cysts.

## Material and Methods

- Protocol and registration

This systematic review was performed according to the Preferred Reporting Items for Systematic Reviews and Meta-analyses (PRISMA) statements (23). The protocol for this systematic review was registered at the International Prospective Register of Systematic Reviews (PROSPERO) database, under the number CRD 42020189349 (http://www.crd.york.ac.uk/PROSPERO).

- Study design and eligibility criteria

Using the Population, Exposure, Comparator, and Outcome (PECO) strategy to define the eligibility criteria, the present research aimed to answer the following question: “Is the computer-aided diagnosis of odontogenic cysts in digital images of histological tissue sections reliable?”

Only studies that evaluated the accuracy of computer-aided histopathological diagnosis of odontogenic cysts were included. No language or publication year were imposed. The following exclusion criteria were applied: studies in which the subject of interest was not addressed, abstracts or indexes, literature reviews, case reports, personal or short communications, book chapters, and patents.

- Search and information sources

Literature search was performed in December 2019, and updated in September 2020. In order to identify relevant studies, a systematic search was conducted in the following electronic databases: PubMed (including MedLine), Scopus, Web of Science, IEEE Xplore, LIVIVO, SciELO, Embase, and LILACS. A partial grey literature search was performed through OpenGrey, OpenThesis, and Google Scholar. A manual search of reference lists from articles retrieved in the initial search strategy was also conducted, for the purpose of identifying additional studies that could not be located in the electronic databases. These procedures were performed to avoid potential selection or publication biases.

Descriptors were selected using the Descriptors in Health Science (DeCS), the Medical Subject Headings (MeSH) and the Embase Subject Headings (Emtree). Boolean operators (AND and OR) were used to combine descriptors and improve the search strategy by means of different combinations. The full electronic search strategy is illustrated in Table 1. All references obtained from PubMed, Scopus, Web of Science, IEEE Xplore, LIVIVO, SciELO, Embase, and LILACS were exported to EndNote Web™ (Thomson Reuters™, Toronto, Canada) software, in which duplicated records were removed. Additionally, the remaining references and studies retrieved from OpenGrey, OpenThesis, and Google Scholar were exported to Microsoft Word™ 2016 (Microsoft™ Ltd., Washington, USA) software, with a view to manually remove duplicates.

**Table 1:**
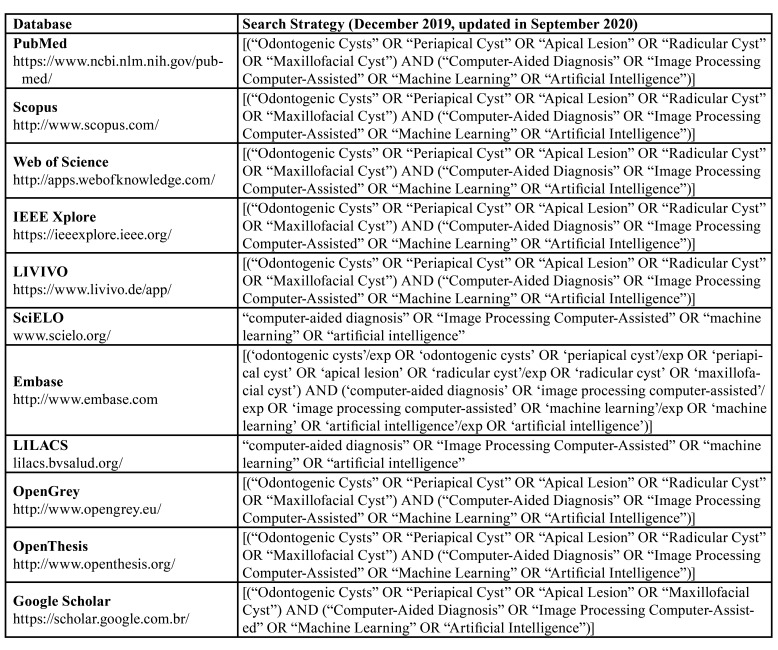
Electronic databases and applied search strategy.

- Study selection

The data collection was independently performed by two reviewers (PHSM and MAVB), in three different phases. First, reviewers discussed the eligibility criteria and applied them to 20% of the studies, aiming to assess potential errors in the method. The inter-rater concordance was evaluated by the Kappa statistic, obtaining a strong agreement (K ≥ 0.81) and confirming the reproducibility and reliability of the evaluation. Then, titles were carefully read to exclude articles out of the scope of this research. Reviewers were not blinded for authorship or name of the journals. Studies in which the subject of interest could not be addressed were excluded.

In phase 2, abstracts were independently analyzed by the two reviewers. At this stage, abstracts in which the subject of interest could not be addressed, congress abstracts, book chapters, case reports, patents, and literature reviews were excluded.

In phase 3, articles had their full-texts evaluated, and their reference lists carefully read in order to identify studies that could not be located in the initial search. Then, articles were assessed to verify whether they fulfilled all eligibility criteria. Articles in which diagnosis of odontogenic cysts was not performed with the aid of computer image processing were excluded. When mutual agreement between the two reviewers was not reached, a third reviewer (LRP) was involved to make a final decision. Excluded studies and reasons for their exclusion were recorded.

- Data collection process and data items

After the screening was done, texts of selected articles were reviewed and data were extracted in a systematic way, including authorship, year of publication, and country of origin of the article; information on the study sample (number of samples, type of lesion, and number of cases); information on the image processing features (type of exam, segmentation method, extracted features); and the way the results were achieved (classification method, validation method and accuracy).

In order to ensure consistency among reviewers, a calibration exercise was performed with both reviewers (PHSM and MAVB), in which information were extracted jointly from an eligible study. One author (PHSM) extracted all the above-mentioned information and the second author (MAVB) cross-checked it to confirm the concordance of data extracted. Any disagreements between the reviewers were solved by discussion with a third author (LRP). When additional assistance was necessary to make a final decision, a fourth author (CB) was consulted.

- Risk of bias in individual studies

The risk of bias of the selected articles was investigated using the Joanna Briggs Institute (JBI) Critical Appraisal tool for use in JBI systematic review studies involving diagnostic accuracy (24). Following the PRISMA recommendations (23), each domain related to the potential risk of bias was independently evaluated by two reviewers (MAVB and LRP). The following questions were used for this evaluation: “(1) was the study based on a consecutive or random sample?; (2) was a case control design avoided?; (3) did the study avoid inappropriate exclusions?; (4) were the index test results interpreted without knowledge of the results of the reference standard?; (5) if a threshold was used, was it pre-specified?; (6) is the reference standard likely to correctly classify the target condition?; (7) were the reference standard results interpreted without knowledge of the results of the index test?; (8) was there an appropriate interval between index test and reference standard?; (9) did all patients receive the same reference standard?; (10) were all patients included in the analysis?” (24). Then, the risk of bias would be rated as high when the study reached up to 49% score “yes”, moderate when it reached 50% to 69% score “yes”, and low when it reached more than 70% score “yes”.

- Data analysis

The heterogeneity among the included articles was analyzed through the examination of the study characteristics, such as dissimilarity among the lesion types, epithelium segmentation, extracted features, classification methods, and outcomes of interest (25). A meta-analysis was planned, considering that data from included articles were relatively homogeneous and appropriate for pooling. If data were heterogeneous and inappropriate for a meta-analysis, a descriptive analysis and summary of the main findings of the selected studies would be performed.

## Results

- Study selection

**Figure 1:**
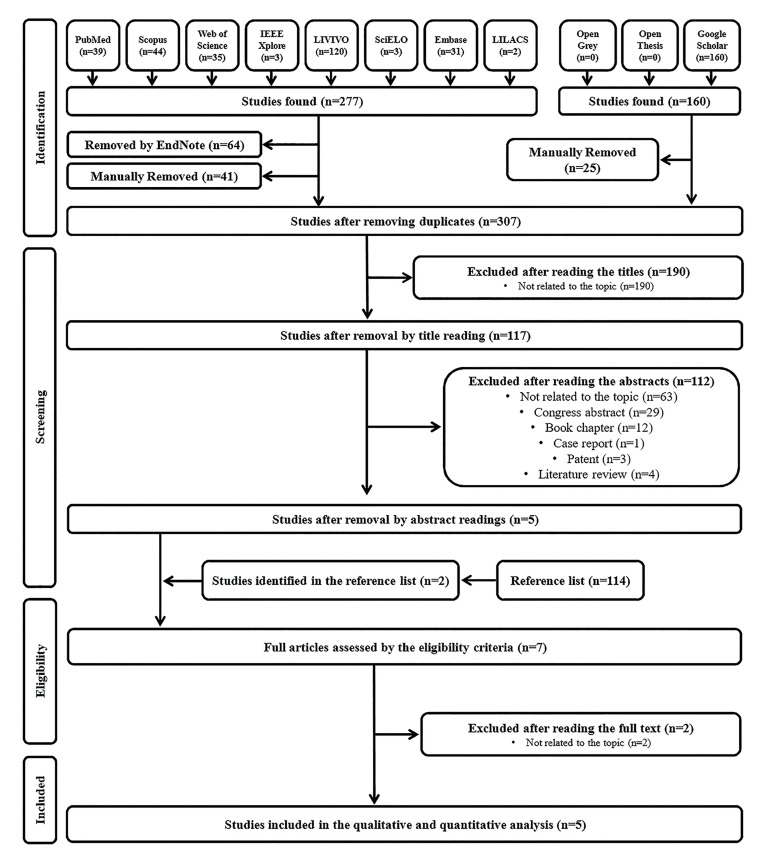
Flowchart showing the results of the search process.

After the systematic search performed within 11 electronic databases, 437 studies were retrieved, of which 130 were duplicates. After removing duplicates, 307 references had their titles carefully read. A total of 190 references were excluded since they did not fulfill the eligibility criteria. Then, in phase 2, 117 studies had their abstracts analyzed and 112 were excluded. In phase 3, the remaining five articles (9,18,20-22) were selected for full-text reading and assessment of the eligibility criteria. Their reference lists were also carefully read for the purpose of identifying studies that were not located in the original search. After reading their reference lists, two new titles were selected (19,26). After reading the full-texts of the seven articles, the study by Wiener *et al*. (26) was excluded because the diagnosis of odontogenic cysts was based on clinical and radiological findings. Furthermore, the study by Eramian *et al*. (9) was excluded because authors only presented an algorithm for the automated segmentation of the epithelial layer of odontogenic cyst tissue. Finally, five articles were included both in qualitative and quantitative analysis (18-22). A flowchart depicting the selection process based in the PRISMA diagram (23) is provided in Fig. 1.

- Characteristics of the selected studies

Selected articles were published between 2006 and 2019, all of them written in English (18-22). Studies were conducted by research groups from four different countries, namely United Kingdom (18,19), Canada (20), Brazil (21), and Japan (22). Table 2 provides a summary of their characteristics.

**Table 2:**
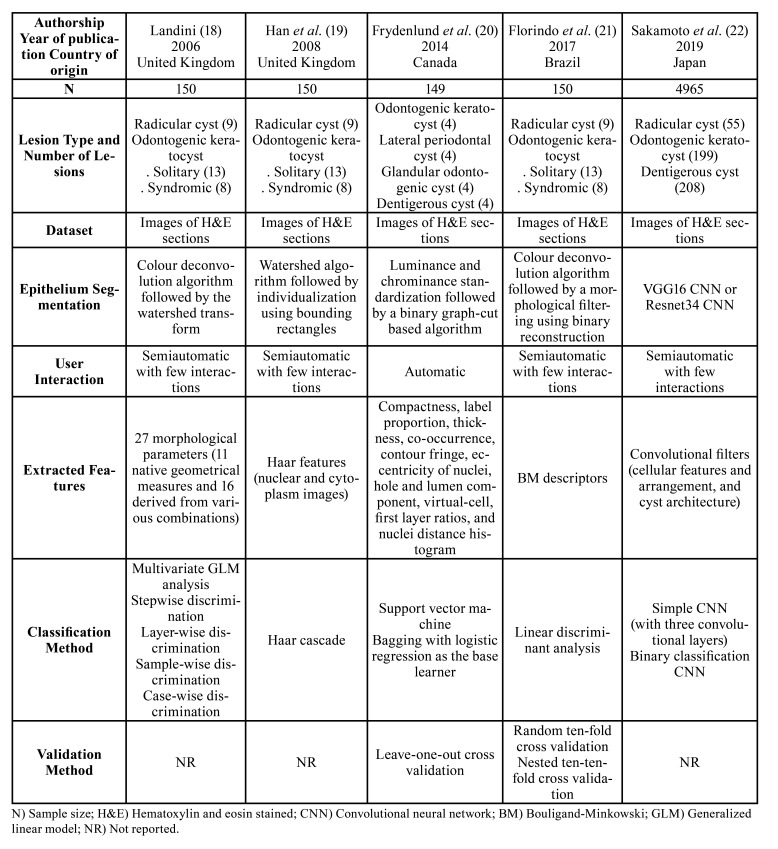
Characteristics of studies included in the systematic review.

Just one out of the five eligible studies mentioned about the approval by the ethical committee (22). Computer-aided diagnosis was performed using images of hematoxylin and eosin sections from formalin fixed and paraffin embedded specimens of odontogenic cysts in all selected studies (18-22). Altogether, 5,264 images from 508 patients were analyzed. In three articles (18,19,21), lesions were classified by the CAD system as radicular cyst or odontogenic keratocyst, the latter considering its subtypes sporadic or syndromic, and compared to previous diagnoses made by specialists. One study classified lesions as odontogenic keratocyst, lateral periodontal cyst, glandular odontogenic cyst or dentigerous cyst (20), while the other as radicular cyst, odontogenic keratocyst or dentigerous cyst (22).

Different methods of epithelium segmentation were used. As it is not possible to consistently define, under light microscopy, the limits between adjacent epithelial cells, a semiautomated space partition procedure based on the colour deconvolution algorithm and the watershed transform was performed in one study (18). Bounding rectangles after information acquired by a prior watershed algorithm were applied in other one (19), and a combination of colour deconvolution and morphological filtering, using binary reconstruction, was used in a third one (21). The VGG16 or Resnet34 convolutional neural networks were fine-tuned to the dataset of one article (22). With the aim of reducing luminance and chrominance variation between images, Frydenlund *et al*. (20) colour-standardized the dataset using an algorithm for automated epithelium segmentation, based on binary graph cuts, proposed by Eramian *et al*. (9).

Morphometrical parameters were the main features extracted for cyst classification in most of the articles. One study considered 27 morphological features, including the longest axis of the cell (18), called Feret diameter, and its angle of orientation. In another article (19), images of lining epithelial cells subdivided into individual nuclear/cytoplasm sub-images were acquired. A total of 52 epithelial description features, grouped as compactness, label proportion, thickness, co-occurrence, contour fringe, eccentricity of nuclei, largest hole and lumen component, virtual-cell, first layer ratios, and nuclei distance histogram were captured in one study (20). Florindo *et al*. (21) used the Boulingand-Minkowski curve as a representation of the image homogeneity at various scales and it has made it appealing for characterization of image complexity. With this method, they achieved 64 descriptors, although only the first 50 were used, as no significant classification gain was observed beyond this number. Finally, in one study (22), convolutional filters were used to assess the cellular features and their arrangement, as well as the lesion architecture.

The eligible studies used different classification methods for the cysts based on the extracted features. The multivariate generalized linear model (GLM) analysis, followed by a hierarchical stepwise discriminant analysis, a layer-wise discriminant analysis, and then a sample and case-wise discriminant analyses were used in one article (18). The Haar cascade classifier, successfully applied to solve some challenging computer tasks, was applied in another one (19). Other two classifiers, a support vector machine (SVM) and an ensemble method bagging with logistic regression as the base learner, were considered in one research (20). In one study (21), the linear discriminant analysis (LDA) supervised classifier was used. Sakamoto *et al*. (22) employed a simple convolutional neural network (CNN) with three layers to investigate the cyst architecture and classify the image, whose output was then used to train another CNN for a binary classification.

Three of the eligible articles have not mentioned the use of any validation methods for verification of the classifier performance (18,19,22). The leave-one-out cross validation (LOOCV) was employed in one article (20), while in the other (21), two approaches were used, the random ten-fold and the nested ten-ten-fold cross validations. The first method evaluated the performance of the classifier when the number of descriptors varied. The second differed in that, instead of running over all possible numbers of features, it selected an ideal number to be used. In this way, an external fold was responsible for the classification and an internal one was used to determine the optimum number of descriptors.

All studies analyzed their system performance through accuracy measurements (18-22). In two of them (19,20), the positive and negative predictive values were used to describe the performance of the different classifiers. In addition to the sensitivity, precision, and specificity values, Sakamoto *et al*. (22) visually assessed the diagnostic ability of the classifier system using the receiver operating characteristic (ROC) curve.

- Risk of bias within the studies

The methodological risk of bias evaluation using the Joanna Briggs Institute (JBI) Critical Appraisal tool which is used in JBI systematic review studies involving diagnosis accuracy (24) is shown in Table 3, where it is possible to depict the answers to the ten questions. None of the included articles fulfilled all the criteria from the checklist. All studies scored low risk of bias (18-22). Question 4 was answered as “no” because histopathological diagnosis provided by pathologists was already known before image processing, and question 8 was considered “unclear” because no study defined the interval between histopathological exams and computer-aided diagnosis.

- Results of individual studies

The overall evaluation showed that all studies presented high accuracy rates of computer image processing methods in diagnosing odontogenic cysts. Although additional types of cysts have been evaluated in two studies (20,22), odontogenic keratocysts, followed by radicular cysts, were the preferred of authors for obtaining the accuracy measures, since they were evaluated in five and four, respectively, of the five eligible studies.

In Landini’s study (18), for all referred classifiers, the best performance was achieved when the two subtypes of odontogenic keratocysts (solitary or syndromic) were pooled together. The individual classifiers with highest accuracy were the sample-wise and the case-wise discriminant analyses, resulting in performances of 95% and 96.7% success rates, respectively, for discriminating odontogenic keratocysts and radicular cysts.

The proposed Haar cascade classifier, used by Han *et al*. (19), was reasonably successful in locating individual cells/nuclei, and cysts were correctly classified as odontogenic keratocysts or radicular cysts in a rate of 86%. The classification rate between the two subtypes of odontogenic keratocysts (solitary or syndromic) was 53.8% and seems to show that there are no characteristic morphological differences between them.

**Table 3:**
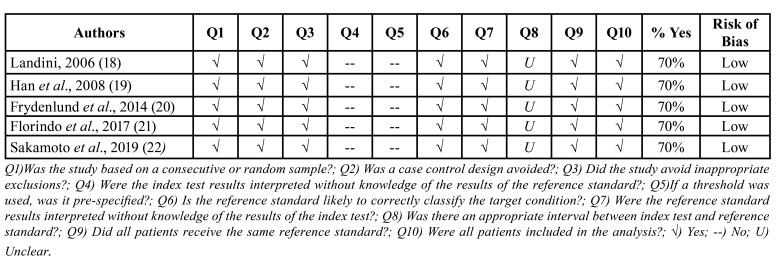
Risk of bias assessed by the Joanna Briggs Institute (JBI) Critical Appraisal tool for use in JBI systematic review studies involving diagnosis accuracy.

The two classifiers used by Frydenlund *et al*. (20) had their parameters optimized with the aim of obtaining better performance. Using bagging with logistic regression as the base learner, the type of odontogenic cyst, if keratocyst, lateral periodontal, glandular, or dentigerous, was correctly diagnosed in a rate of 90% to 95.4%. The support vector machine achieved a slightly lower result, on average, ranging between 83.8% and 92.3%. One particular cyst type, the dentigerous cyst, was associated with the majority of misclassifications. Omission of this cyst from the dataset improved the classification rate for the remaining three cyst types to 96.2% for both classifiers.

Florindo *et al*. (21) used the Boulingand-Minkowski descriptors for image classification as input parameters of a linear discriminant analysis supervised classifier associated with the nested cross validation method. They were capable of discriminating between odontogenic keratocysts and radicular cysts in 98% of images. When the two subtypes of odontogenic keratocysts (solitary or syndromic) were considered, the classification rate in the discrimination between the three types of cysts was 72%. When discriminating between the two subtypes of odontogenic keratocysts, the accuracy rate was 68%. The case-wise analysis showed slightly better success rates of 100% for odontogenic keratocysts and radicular cysts, 76% for the three types of cysts, and 71% for the two subtypes of odontogenic keratocysts.

Finally, at the remaining selected article (22), although three types of odontogenic cysts (radicular, keratocyst, and dentigerous) have been studied, the individual classifier with highest accuracy was the binary convolutional neural network (CNN) trained for keratocyst or non-keratocyst classification, achieving a performance of 99.8% success rate, when the VGG16 CNN was used, and of 99.6% when using the Resnet34 CNN.

- Synthesis of results

Although all studies have investigated the computer-aided histopathological diagnosis of odontogenic keratocysts and, in four of them, of radicular cysts, they have described different types of classification methods for the lesions based on the extracted features. In addition, only two studies have mentioned the use of validation methods to validate the classification experiments. Therefore, because of differences in the study designs and in the collected information, the pooled data (meta-analytical approach) from the selected studies were deemed not suiTable.

## Discussion

This systematic review aimed to evaluate the accuracy of computer image processing for performing the diagnosis of odontogenic cysts in comparison to the diagnosis previously determined by histopathologists using both histological and clinical information from the respective patients. In recent years, many attempts have been made to use automated machine vision systems for the analysis of medical images (27), including breast cancer (28) and colorectal cancer (29), which affect a large number of people around the world. However, each study considers a specific method depending on the characteristics of the disease of interest. In head and neck areas, only few published articles exist that address computer-aided detection and classification of lesions related to dental structures (8,17,30). A recent systematic review and meta-analysis evaluated the accuracy of the novel approaches and demonstrated the effectiveness of CAD systems in classifying lesions based on cone beam computed tomography images (31). When digital analysis of odontogenic cysts is performed using microscopy images, especially for discriminating odontogenic keratocysts and radicular cysts, a variation of 86% to 100% was observed between the least and most accurate classification methods of the eligible studies, indicating a very promising future for an automated cyst class prediction.

In 2006, Landini (18) described a quantitative analysis of the epithelial lining architecture in digitized histological images of radicular cysts and odontogenic keratocysts, which were segmented into theoretical cells using a semi-automated partition based on the intensity of the hematoxylin stain. Various morphometrical parameters were extracted from those cells and epithelial layer membership was computed using a systematic clustering routine. This mathematically modeled approach provided an extra level of hierarchical description that the individual cell morphology alone could not provide, allowing a high rate of discrimination between radicular and odontogenic keratocyst linings, 96.7% using the classifier with highest accuracy, the case-wise discriminant analysis. However, differences between solitary and syndromic keratocysts did not allow proper diagnosis of these subtypes.

Two years later, with the rapid development of machine learning algorithms, Han *et al*. (19) used the Haar cascade classifier, a cascade of boosted classifiers applying Haar-like features, to explore the feasibility of using such algorithms to classify the same types of odontogenic cysts. Despite they have gathered the data from the same sample of a previous research, radicular cysts and odontogenic keratocysts were correctly classified in a rate of 86%, there have been many false positive detections. This performance reflects the fact that this technique was based on lesser information, while the methods of Landini (18) considered tissue structural properties in addition to information on individual cells to obtain a high overall correct classification result.

Digital micrographs of lateral periodontal cysts, glandular odontogenic cysts, and dentigerous cysts, in addition to odontogenic keratocysts, were also evaluated in the research of Frydenlund *et al*. (20). In their study, they combined properties of Landini’s (18) theoretical cells with morphological, spectral, and textural features of the entire visible epithelial region. An important step towards the computer-assisted diagnostic system for classifying odontogenic cysts was the automated epithelial segmentation algorithm proposed by Eramian *et al*. (9). Then, Frydenlund *et al*. (20) presented a second step towards a fully automated algorithm. They proposed a set of image features that could be computed from such epithelial regions to form descriptions of the regions and evaluated its effectiveness by using two statistical classifiers, a support vector machine and bagging with logistic regression as the base learner. With the latter, the type of odontogenic cyst was correctly diagnosed in a rate of 90% to 95.4%. In addition, this success rate was improved to about 96.2% with omission of dentigerous cysts from the dataset, associated with the majority of misclassifications.

Considering that these previous studies (18,19,20) investigated only basic feature-based methods, Florindo *et al*. (21) tried to use a different approach to assess the classification of cyst lining images, based on a machine vision algorithm, the Bouligand-Minkowski fractal descriptors. These did not require the level of abstraction necessary to extract histological relevant features and had the potential of being more robust than those previous approaches. The descriptors were obtained by mapping pixel intensities into a three-dimensional cloud of points in discrete space and applying morphological dilations with spheres of increasing radii. The descriptors were computed from the volume of the dilated set and submitted to a machine learning algorithm to classify the samples into diagnostic groups. They used the same sample of Landini (18) and Han *et al*. (19), and achieved better performances. The patient-wise analysis had success rates of 100% for discriminating between odontogenic keratocyst and radicular cyst, 71% for the two subtypes of odontogenic keratocysts, and 76% for the three types of cysts, confirming that the discrimination between keratocysts is a more difficult challenge than the identification of radicular cysts. These results also suggest that in addition to histological relevant structures, such as those used in previous studies, pixel intensities also carry useful diagnostic importance for image characterization, highlighting the value of texture-based image analysis to describe complex histological images.

Recently, a great interest has been focused on the deep learning-based methods, which have been extensively used for solving complex problems in medical radiology (32). Deep learning can overcome some limitations of other conventional methods, and the most established deep learning algorithm among various models is the convolutional neural network (CNN), a class of artificial neural networks that has been a dominant method in computer vision tasks, including various diagnostic tasks (33). Since algorithms used in previous studies were conventional feature engineering algorithms, optimized and tested using a relatively small number of samples (18,19,21), a deep learning-based approach that have used a larger dataset could improve the precision of the classification process. Using a sample of almost 5000 microscopy images obtained from 55 radicular cysts, 199 odontogenic keratocysts, and 208 dentigerous cysts, Sakamoto *et al*. (22) tried a strategy of applying two CNNs. The first one investigated the cellular features and their arrangement, while the second one investigated the cyst architecture and assigned a class to the entire image. However, as mentioned before, the individual classifier with highest accuracy was the binary CNN trained for keratocyst and non-keratocyst classification, which achieved a performance of 99.8% success rate when VGG16 CNN was used. While still an incipient method for histopathological diagnosis, they concluded that a straightforward solution for ensuring that the neural network learns these specific histological characteristics would be to annotate the images with the particular features as subclasses, and that further studies are needed to determine whether CNNs can handle these ambiguous histological features.

One important point that should be highlighted is the small variety of lesions addressed in the eligible studies. Similar CAD systems have been widely and successfully applied in many medical areas, providing physicians with a valuable tool. However, despite the commonality of dental procedures, the field of dentistry has not yet fully benefited from the advancements of these technologies, this fact being reflected in the low number of studies eligible for this systematic review. In addition, all datasets were composed by images of hematoxylin and eosin sections. However, there are other methods now available that are probably more suiTable for machine vision. The use of a simple nuclear staining technique such as DAPI staining for accessing the cell pattern, or the use of immunostaining with anti-keratin antibody for visualizing the cyst architecture are examples. Then, it remains a challenge to develop an image analysis system that can mimic the performance of pathologists in diagnosing these lesions. Future studies, with well-designed methodologies, including standardized and reproducible methods of segmentation and analysis, are encouraged.

## Conclusions

All individual studies selected for this systematic review suggested the great potential of CAD system for accurately classifying odontogenic cysts, especially radicular cysts and odontogenic keratocysts, compared to previous diagnosis performed by histopathologists. However, due to the heterogeneity of studies, a meta-analysis evaluating the outcomes of interest was not performed, and a pragmatic recommendation about its use is not possible. Further standardized studies are needed to increase the strength of evidence and to confirm the accuracy of computer-assisted analysis for diagnosing all types of odontogenic cysts.
